# Antioxidant Natural Compounds Integrated with Targeted Protein Degradation: A Multi-Modal Strategy for Alzheimer’s Disease Therapy

**DOI:** 10.3390/antiox14121426

**Published:** 2025-11-27

**Authors:** Desh Deepak Singh, Dharmendra Kumar Yadav, Dongyun Shin

**Affiliations:** 1 Amity Institute of Biotechnology, Amity University Rajasthan, Jaipur 303002, India; ddsbms@gmail.com; 2 College of Pharmacy, Gachon University, Hambakmoeiro 191, Yeonsu-gu, Incheon 21924, Republic of Korea

**Keywords:** Alzheimer’s disease, antioxidant natural compounds, targeted protein degradation, PROTACs, tau protein, amyloid-β, oxidative stress, neuroprotection

## Abstract

Alzheimer’s disease (AD) Alzheimer’s disease (AD) is a progressive neurodegenerative disorder marked by protein aggregation, oxidative stress, mitochondrial dysfunction, and chronic neuroinflammation, leading to cognitive decline. Current therapies remain largely symptomatic, highlighting the need for multi-target therapeutic strategies. Recent advances in antioxidant natural compounds and targeted protein degradation (TPD) technologies—particularly proteolysis-targeting chimeras (PROTACs), offer complementary mechanisms for disease modification. Natural antioxidants, including flavonoids, polyphenols, terpenoids, and alkaloids, confer neuroprotection by reducing reactive oxygen species, activating Nrf2 pathways, restoring mitochondrial function, and suppressing neuroinflammation. PROTACs, in contrast, selectively degrade pathological proteins such as hyperphosphorylated tau, amyloid-β, and APP fragments through the ubiquitin–proteasome system. The integrated “Antiox-PROTAC” approach combines these modalities to simultaneously mitigate oxidative stress and eliminate neurotoxic proteins. Natural compounds may act as warheads or scaffolds in PROTAC design, retaining antioxidant activity while enabling targeted degradation. Early preclinical findings demonstrate synergistic neuroprotective potential, though translational challenges remain, including blood–brain barrier permeability, bioavailability, and delivery optimization. Future directions involve hybrid molecules, nanoparticle-based delivery, and personalized therapeutic strategies. Overall, the Antiox-PROTAC paradigm represents a next-generation, multi-modal framework with the potential to modify disease progression and enhance cognitive outcomes in Alzheimer’s disease.

## 1. Introduction

Alzheimer’s disease (AD) is the most prevalent neurodegenerative disorder, accounting for 60–70% of dementia cases worldwide, and represents a major global health challenge in the 21st century [1]. With increasing life expectancy, AD prevalence continues to rise, imposing significant medical, social, and economic burdens [2]. Pathologically, AD is defined by extracellular amyloid-β (Aβ) plaques, intracellular neurofibrillary tangles composed of hyperphosphorylated tau, oxidative stress, neuroinflammation, and synaptic degeneration [2]. Despite decades of research, few disease-modifying therapies exist; most approved treatments provide only symptomatic relief [3]. This limitation underscores the urgent need for innovative, multi-target approaches that act on molecular mechanisms underlying disease progression [4].

Oxidative stress is a central pathological hallmark of AD. Excessive production of reactive oxygen species (ROS) and impaired antioxidant defenses lead to mitochondrial dysfunction, lipid peroxidation, protein oxidation, and neuronal apoptosis [5]. Natural antioxidants—including polyphenols, flavonoids, alkaloids, and terpenoids—exert neuroprotective effects by scavenging ROS, activating Nrf2-mediated antioxidant pathways, restoring mitochondrial homeostasis, and modulating inflammatory signaling [6]. However, clinical translation remains challenging due to limited bioavailability, poor blood–brain barrier (BBB) permeability, and metabolic instability [7]. Enhancing antioxidant efficacy through novel formulations, nanocarriers, or molecular targeting strategies could improve therapeutic outcomes in AD [8].

In parallel, targeted protein degradation (TPD) has emerged as a transformative therapeutic concept. Among these, proteolysis-targeting chimeras (PROTACs) are bifunctional molecules that recruit specific proteins to E3 ubiquitin ligases, leading to their ubiquitination and degradation via the proteasome pathway [9,10]. Unlike traditional enzyme inhibitors, PROTACs act catalytically, allowing sub-stoichiometric dosing and the ability to degrade previously “undruggable” targets, including tau, amyloid precursor protein (APP) fragments, and α-synuclein [11,12]. Preclinical studies have demonstrated successful tau degradation using brain-permeable PROTACs, offering proof-of-concept for their potential in neurodegenerative diseases [12]. Nonetheless, challenges persist—particularly BBB penetration, target selectivity, pharmacokinetic optimization, and off-target degradation, which collectively limit their current clinical translation in AD [13].

Integrating natural antioxidants with PROTAC technology offers a novel and synergistic therapeutic opportunity. Natural compounds possess intrinsic antioxidant, anti-inflammatory, and anti-amyloidogenic properties that can complement the targeted degradative action of PROTACs [14]. When combined, these two modalities—termed “Antiox-PROTACs”—could achieve dual benefits: (1) reduction in oxidative and inflammatory stress, and (2) degradation of neurotoxic proteins that drive disease progression [15]. Moreover, antioxidant natural products may serve as structural scaffolds or ligands in PROTAC design, providing favorable pharmacological properties, improved BBB permeability, and enhanced safety profiles [16]. This integration bridges natural product pharmacology with chemical biology, potentially overcoming limitations inherent in each approach when used alone [17].

This review aims to explore the therapeutic convergence between antioxidant natural compounds and TPD technologies within the context of AD. We first examine the mechanistic role of oxidative stress in AD pathogenesis and summarize major antioxidant classes with neuroprotective potential [18]. Next, we discuss advances in PROTAC-mediated protein degradation, highlighting emerging applications for tau and Aβ clearance in neurodegenerative models [19]. Finally, we propose integration models that combine antioxidant scaffolds with PROTAC platforms to generate multifunctional therapeutic molecules, addressing existing gaps in drug delivery, selectivity, and efficacy [20].

The combination of natural product-based discovery and cutting-edge proteomics exemplifies a paradigm shift toward precision, multi-target AD therapy. The development of hybrid “Antiox-PROTACs” aligns with precision medicine strategies, potentially enabling selective modulation of multiple disease pathways—oxidative damage, protein aggregation, mitochondrial dysfunction, and inflammation—within a single therapeutic entity [21]. Continued innovation in BBB-penetrant delivery systems, rational design, and structure–activity optimization may yield disease-modifying outcomes that surpass conventional monotherapies. Collectively, this integrative framework holds promise to reduce the global burden of Alzheimer’s disease and improve patient quality of life.

## 2. Pathology of Alzheimer’s Disease

**Figure 1:**
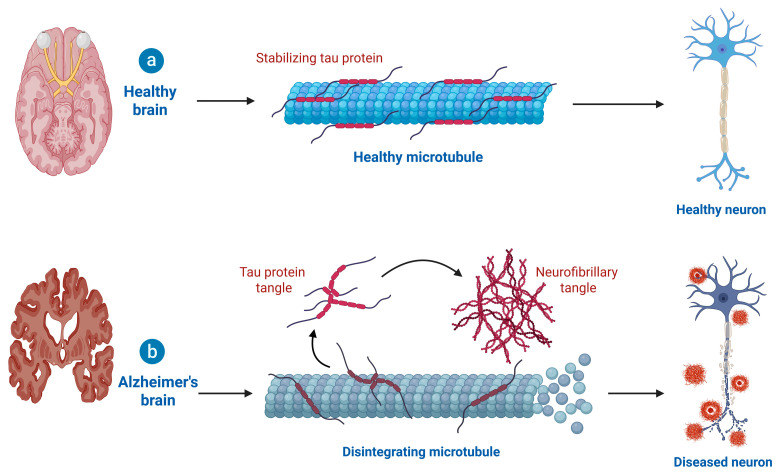
Comparison of tau protein function in healthy and Alzheimer’s brains. (**a**) In a healthy brain, tau proteins stabilize microtubules, maintaining proper neuronal structure and transport. The microtubules remain intact, supporting healthy neuron function. (**b**) In Alzheimer’s disease, tau proteins become hyperphosphorylated and detach from microtubules, leading to their disintegration. The free tau proteins aggregate into neurofibrillary tangles, disrupting neuronal transport and contributing to neurodegeneration (Created in BioRender. Singh, D. (2025) https://BioRender.com/pzlt3uv; accessed on 29 October 2025).

The defining pathological features of Alzheimer’s are neurofibrillary tangles (NFTs) within neurons and amyloid-beta (Aβ) plaques extracellularly. Recent evidence has shown that small soluble intracellular amyloid-β (Aβ) oligomers, rather than the large extracellular plaques, represent the most neurotoxic species of Aβ. These oligomers disrupt synaptic signaling, impair mitochondrial function, and induce oxidative stress and neuroinflammatory responses, leading to progressive neuronal dysfunction. Aβ is a peptide generated by the abnormal sequential cleavage of amyloid precursor protein (APP) by β- and γ-secretases, resulting in peptides that aggregate within and around neurons. While extracellular accumulation of Aβ interferes with neuronal communication and activates microglia through excitotoxic and inflammatory pathways, hyperphosphorylated tau, a microtubule-associated protein, self-aggregates into paired helical filaments that form neurofibrillary tangles (NFTs), causing cytoskeletal destabilization, axonal transport defects, and neuronal apoptosis. Together, these pathological processes contribute synergistically to the neurodegenerative cascade observed in Alzheimer’s disease (Figure 1) [22]. The amalgamation of the toxic mechanisms from the Aβ and tau pathology leads to an eventual cascade of synaptic degeneration, impaired mitochondrial function, and disrupted calcium homeostasis and cognitive decline [23]. Furthermore, neuroinflammation is notably important as activated microglia and astrocytes release pro-inflammatory cytokines, reactive oxygen species, and activated glial cells can contribute to neuronal injury [24]. Vascular contributions to pathology, including cerebral amyloid angiopathy, can limit cerebral blood flow and compromise the blood–brain barrier and further pathology. Genetic risk factors, particularly mutations in APP and presenilin 1 and 2 (PSEN1, PSEN2), and the presence of the apolipoprotein E ε4 (APOE ε4) allele increase risk through their effects on the metabolism and clearance of Aβ [25]. Other important pathogenic mechanisms include cholinergic neuronal loss in the basal forebrain, loss of neurotransmitters (e.g., loss of acetylcholine), glutamate dysregulation, and synaptic vesicle dysfunction, which all contribute to memory and learning [26]. Over time, neuronal death results in observable brain changes such as cortical thinning, ventricular enlargement, and medial temporal lobe atrophy that can be seen on neuroimaging [27]. The pathological process of Alzheimer’s disease (AD) emerges years before observable clinical signs, often progressing through mild cognitive impairment to more advanced stages of dementia, including severe dementia [28]. While AD is the leading cause of dementia worldwide, the specifics of its development are still relatively unclear, although several aspects of the disease are multifactorial in nature, with evidence supporting possible involvement from genetic, environmental, vascular, and lifestyle mechanisms [29]. The final pathology of AD then consists of all of the aforementioned processes converging with amyloid deposition, tau aggregation, neuroinflammation, vascular dysfunction, and neurotransmitter deficiency contributing to an ongoing breakdown in cognitive, functional, and behavioral capacity [30].

## 3. Antioxidant Natural Compounds in Neuroprotection

### 3.1. Oxidative Stress and Mitochondrial Dysfunction in AD

**Figure 2:**
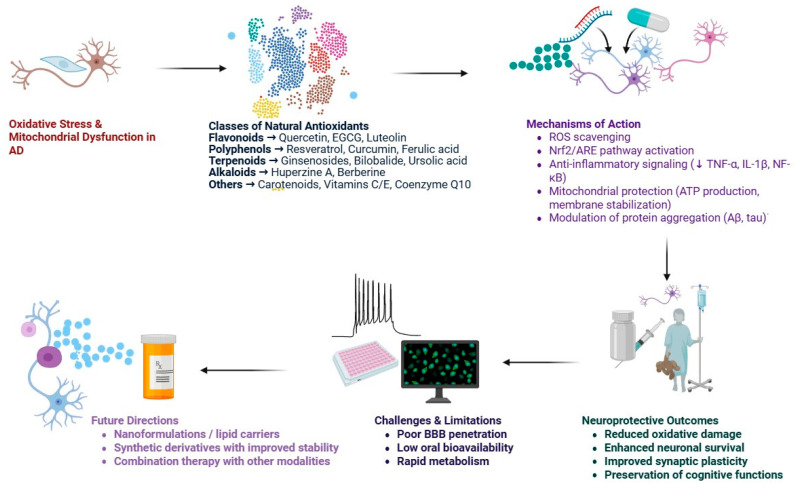
Role of natural antioxidants in Alzheimer’s disease (AD). Oxidative stress and mitochondrial dysfunction play central roles in AD pathogenesis. Natural antioxidants—including flavonoids (quercetin, EGCG, luteolin), polyphenols (resveratrol, curcumin, ferulic acid), terpenoids (ginsenosides, bilobalide, ursolic acid), alkaloids (huperzine A, berberine), and others (carotenoids, vitamins C/E, coenzyme Q10)—exert neuroprotective effects via multiple mechanisms. These include ROS scavenging, Nrf2/ARE pathway activation, anti-inflammatory signaling (↓ TNF-α, IL-1β, NF-κB), mitochondrial protection (ATP production, membrane stabilization), and modulation of protein aggregation (Aβ, tau). Neuroprotective outcomes include reduced oxidative damage, enhanced neuronal survival, improved synaptic plasticity, and preservation of cognitive functions. However, challenges such as poor blood–brain barrier penetration, low oral bioavailability, and rapid metabolism limit their therapeutic potential. Future strategies involve nanocarriers/lipid formulations, synthetic derivatives with improved stability, and combination therapy with other modalities. (Created in BioRender. Singh, D. (2025) https://BioRender.com/pzlt3uv; accessed on 29 October 2025).

Oxidative stress is a critical pathological mechanism in Alzheimer’s disease (AD) that contributes to neuronal loss and cognitive decline. Excessive production of reactive oxygen species (ROS), resulting from mitochondrial dysfunction, impaired antioxidant defenses, and neuroinflammatory processes, exacerbates neurodegeneration (Figure 2) [31]. Consistent findings have identified mitochondrial dysfunction in AD brains, including reduced cytochrome oxidase activity, impaired ATP synthesis, and increased lipid peroxidation [32]. These alterations disrupt neuronal energy homeostasis and amplify oxidative damage to proteins, lipids, and nucleic acids. Moreover, Aβ peptides and hyperphosphorylated tau species further aggravate oxidative organelle injury by disturbing mitochondrial homeostasis and altering calcium dynamics, thereby perpetuating the cycle of oxidative stress and mitochondrial impairment. Collectively, this interplay between oxidative stress and mitochondrial dysfunction provides a strong rationale for pharmacologically restoring redox balance using natural antioxidants (Table 1).

### 3.2. Classes of Natural Antioxidants

**Figure 3:**
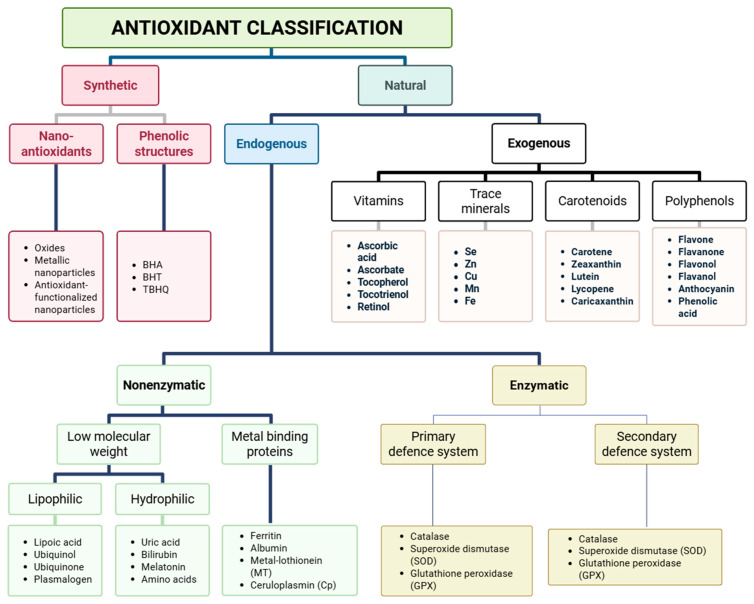
Classification of antioxidants: Antioxidants are broadly classified into synthetic and natural categories. Synthetic antioxidants include nano-antioxidants (oxides, metallic nanoparticles, antioxidant-functionalized nanoparticles) and phenolic structures (BHA, BHT, TBHQ). Natural antioxidants are divided into endogenous and exogenous types. Exogenous antioxidants include vitamins (ascorbic acid, ascorbate, tocopherol, tocotrienol, retinol), trace minerals (Se, Zn, Cu, Mn, Fe), carotenoids (carotene, zeaxanthin, lutein, lycopene, carixanthin), and polyphenols (flavone, flavonol, flavanol, anthocyanin, phenolic acid). Endogenous antioxidants are further classified as nonenzymatic low molecular weight (lipophilic: lipoic acid, ubiquinol, ubiquinone, plasmalogen; hydrophilic: uric acid, bilirubin, melatonin, amino acids) and metal-binding proteins (ferritin, albumin, metallothionein, ceruloplasmin), and enzymatic antioxidants. Enzymatic systems comprise primary defense (catalase, superoxide dismutase [SOD], glutathione peroxidase [GPX]) and secondary defense systems (catalase, SOD, GPX). (Created in BioRender. Singh, D. (2025) https://BioRender.com/pzlt3uv).

Many natural antioxidant compounds exhibit neuroprotective activity in animal models of AD (Figure 3) [39]. This includes dietary antioxidants such as flavonoids, which are present in various foods including fruits, vegetables, and tea and cocoa, and the flavonoid extracts quercetin, epigallocatechin gallate (EGCG), and luteolin demonstrate strong reactive oxygen species (ROS) scavenging activity [40]. Besides their antioxidant properties, flavonoids influence critical tau hyperphosphorylation, as well as target key enzymes implicated in amyloid-β (Aβ) aggregation, influencing two major pathological processes associated with AD [40].

Another group of neuroprotective natural compounds includes polyphenols. Resveratrol, curcumin, and ferulic acid are examples of polyphenols that protect neurons by stimulating mitochondrial biogenesis and intracellular lipid peroxidation and inhibiting pro-inflammatory signaling [41]. Curcumin additionally demonstrates the dual activity of stabilizing and disrupting protein aggregation while reducing oxidative stress. This dual activity may make curcumin an important compound for the future of multi-target therapies aimed at the role of AD [42]. Terpenoids, including compounds like ginsenosides, bilobalide (from Ginkgo biloba), and Ursolic, have demonstrated antioxidative, anti-inflammatory, and neurotrophic effects [43]. These compounds help to maintain the mitochondrial membrane potential, support survival pathways in neurons, and have been shown to improve cognitive outcomes in models of AD, demonstrating their potential in neurodegeneration [44].

Alkaloids, including huperzine A and berberine, demonstrate both antioxidant and neuroprotective effects. Along with their ability to scavenge ROS, these compounds also demonstrate cholinesterase inhibition and increase synaptic acetylcholine levels while reducing neuronal toxicity [45]. This dual mechanism renders alkaloids as particularly interesting options for both symptomatic and disease-modifying interventions in AD. Finally, other natural antioxidants, which include carotenoids, vitamins C and E, and coenzyme Q10, confer neuroprotection through stabilizing cell membranes and supporting mitochondrial functions [46]. These compounds supplement the activities of compounds from the other classes and aid in maintaining cellular homeostasis while under oxidative stress conditions. These classes of natural antioxidants together provide a diverse pharmaceutical arsenal for making multi-target therapeutics aimed at Alzheimer’s disease that ultimately take on challenges related to the complex, multi-faceted nature of neurodegeneration [47].

### 3.3. Mechanisms of Action and ROS Scavenging, Nrf2 Pathway, Anti-Inflammatory Effects

The neuroprotective effectiveness of antioxidant natural compounds is due to their ability to modulate many interrelated cellular pathways, which is especially beneficial in complex diseases such as AD [48]. One major mechanism is the scavenging of reactive oxygen species (ROS), in which some of these compounds act directly to neutralize ROS, thereby decreasing oxidative injury to neuronal lipids, proteins, and DNA [49]. This action prevents the collapse of mitochondria and preserves cellular energy homeostasis, which is disrupted in AD. Another main mechanism is the activation of the Nrf2 pathway [50]. Many of the natural antioxidants activate the nuclear factor erythroid 2–2-related factor 2 (Nrf2), which is a master transcriptional regulator of cellular antioxidant defenses. When Nrf2 is activated, it enhances the expression of cytoprotective genes, including heme oxygenase-1 (HO-1), glutathione peroxidase, and superoxide dismutase, which enhances the neuron’s capability to combat oxidative stress and restore redox balance. Anti-inflammatory effects also aid in a complementary mechanism of neuroprotection [51]. Natural antioxidants modulate microglial activation and inhibit pro-inflammatory mediators, including TNF-α, IL-1β, and NF-κB signaling. By dampening chronic neuroinflammation, the use of antioxidant natural compounds can diminish secondary neuronal injury and suppress the spread of AD pathology [52].

The protection of the mitochondria is another important aspect of a multi-modal or pleiotropic approach [53]. Several antioxidants maintain mitochondrial membrane potential, promote mitochondrial biogenesis, and sustain ATP production in order to avoid the activation of apoptotic pathways. In doing so, not only is neuronal viability maintained, but synaptic function, which is essential for cognition, is also preserved [53]. Finally, several natural compounds directly target protein aggregation, affecting amyloid-β fibrillization and tau hyperphosphorylation. In addition to these direct anti-inflammatory properties, antioxidants act on various upstream and downstream contributors to neurodegeneration by affecting central pathological processes [54]. This multi-modal or pleiotropic character (e.g., antioxidant, anti-inflammatory, mitochondrial, anti-aggregation) makes these natural compounds ideally suited for a multi-target approach in AD pathology [55]. The ability to simultaneously affect several pathways across this disease spectrum underscores their potential as key components in novel therapeutic approaches, such as combination strategies [56].

### 3.4. Challenges of Blood–Brain Barrier (BBB) Permeability and Bioavailability

Despite the encouraging preclinical data discussed earlier, there has been some disappointment in advancing natural antioxidants to clinical application in treating AD [57]. One of the constraints is that natural antioxidants have poor pharmacokinetic properties, such as low solubility, rapid metabolism, and low oral bioavailability [58]. In addition, they must adequately penetrate the blood–brain barrier (BBB) to reach the central nervous system. For example, curcumin and resveratrol display powerful in vitro activity but yield subtherapeutic concentrations in the brain due to the extensive first-pass effect [59]. Polyphenols, as well, undergo rapid glucuronidation and sulfation to limit their systemic availability [60].

To address these challenges, new strategies are being explored. Systems based on nanoparticles, formulations based on liposome delivery, and structural modifications of natural compounds have been explored for improving penetration across the BBB and extending half-life [61]. The conjugation of antioxidant scaffolds to lipid moieties or ligands targeting transporters may further improve CNS delivery [62]. All of these strategies are important to ensure that the neuroprotective potential of natural antioxidants can be translated into clinically relevant neuroprotective therapies [63].

## 4. Targeted Protein Degradation Technologies

### 4.1. Overview of PROTACs, Molecular Glues, and Other TPD Modalities

**Figure 4:**
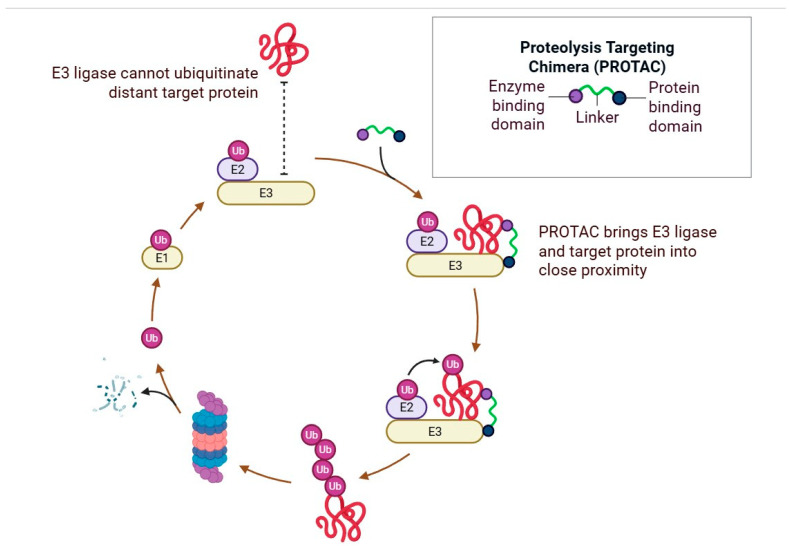
Mechanism of PROTAC-mediated targeted protein degradation. In the absence of PROTAC, E3 ubiquitin ligase cannot ubiquitinate distant target proteins. PROTACs (Proteolysis-Targeting Chimeras) are bifunctional molecules composed of an enzyme-binding domain, a protein-binding domain, and a linker that connects them. PROTACs facilitate the proximity of the E3 ligase and the target protein, allowing ubiquitination of the target protein. The ubiquitinated protein is subsequently recognized and degraded by the proteasome, leading to the selective removal of pathogenic or unwanted proteins (Created in BioRender. Singh, D. (2025) https://BioRender.com/pzlt3uv; accessed on 29 October 2025).

Targeted protein degradation (TPD) represents a novel paradigm shift towards selectively eliminating disease-causing proteins instead of only inhibiting their activities [64]. In the TPD technologies, proteolysis-targeting chimeras (PROTACs) are bifunctional compounds that induce the target protein to an E3 ubiquitin ligase, resulting in ubiquitination and degradation via the proteasome [65]. Importantly, PROTACs act catalytically (Figure 4), allowing for sub-stoichiometric dosing and, unlike small molecules, the ability to target proteins that would have previously been considered “undruggable,” including some associated with neurodegenerative diseases [65]. Other modalities, including molecular glues, stabilize an interaction between an E3 ligase and a target protein, without the need for a linker. Further, with emerging TPD modalities like lysosome-targeting chimeras (LYTACs) and autophagy-targeting chimeras (AUTACs), scientists can harness degradation pathways besides just proteasomal degradation [66]. Collectively, this set of tools affords a global strategy for targeted protein modulation in complex disease landscapes [67].

### 4.2. Main E3 Ligases and the Implications in CNS Disorders

**Figure 5:**
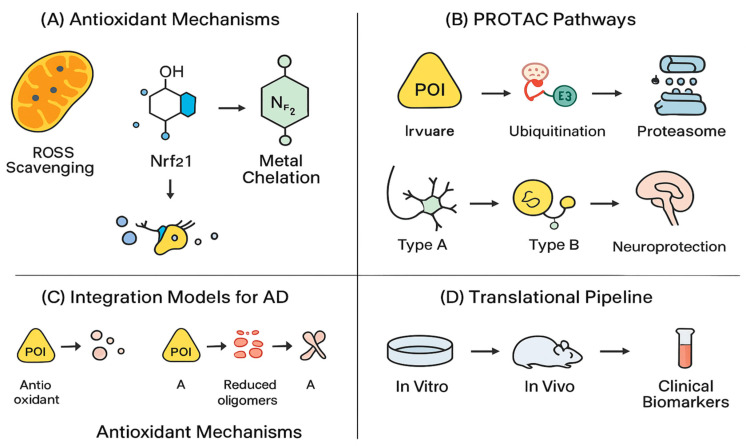
Schematic representation of antioxidant–PROTAC integration strategies for Alzheimer’s disease (AD) therapy. (**A**) Antioxidant mechanisms: scavenging of reactive oxygen species (ROS), activation of the Nrf2 pathway, and metal chelation to mitigate oxidative stress. (**B**) PROTAC pathways: formation of a ternary complex between the protein of interest (POI) and E3 ligase leading to ubiquitination and proteasomal degradation, resulting in neuroprotection. (**C**) Integration models for AD: combining antioxidant and PROTAC mechanisms to reduce amyloid-β (Aβ) oligomers and aggregation. (**D**) Translational pipeline: progression from in vitro and in vivo validation to pharmacokinetic/pharmacodynamic evaluation and clinical biomarker assessment (Created in BioRender. Singh, D. (2025) https://BioRender.com/pzlt3uv; accessed on 29 October 2025).

E3 ubiquitin ligases are pivotal in defining TPD specificity due to their capacity to determine which proteins are recruited for degradation [68]. Some of the most common ligases used in TPD research include cereblon (CRBN), von Hippel–Lindau (VHL), and MDM2, all of which have unique substrate selectivity [69]. When considering CNS disorders, the expression and distribution of the ligases will be important. If the ligase is not adequately distributed in the CNS, then degradation will not work efficiently [70]. Recent evidence has shown E3 ligase profiles in various neuronal and glial populations, providing information on potential candidates for PROTAC therapies for neurodegenerative diseases such as Alzheimer’s disease and Parkinson’s disease (Figure 5) [70]. Integration of Antioxidants and PROTAC Pathways in Alzheimer’s Disease Therapy is shown in Table 2.

### 4.3. Neurodegenerative Applications of PROTACs

The use of PROTACs in neurodegenerative diseases is an emerging area of investigation. Preclinical studies have shown that tau-targeted PROTACs can reduce tau aggregation, one of the pathological hallmarks of AD, and alpha-synuclein-recruited PROTACs have shown potential in animal models of Parkinson’s disease [71]. The ability of PROTACs to selectively degrade the disease-causing protein, rather than just inhibit its function as is observed with most small-molecule inhibitors, provides several distinct advantages, including attenuating toxic gain-of-function properties or decreasing protein accumulation [72]. Natural product-derived scaffolds or CNS-penetrant ligands in bifunctional designs are currently being tested to improve the specificity and bioavailability of the PROTACs in development. Next-generation neurotherapeutics are on the horizon [73].

### 4.4. Challenges of TPD in the Brain

Promising approach to mitigating the extracellular Aβ burden in Alzheimer’s disease. By selectively degrading key Aβ-generating enzymes such as β-secretase (BACE1) or other APP-processing components, PROTACs can effectively reduce Aβ peptide formation [72]. Moreover, targeting intracellular Aβ oligomers for proteasomal degradation may enhance cellular clearance mechanisms, thereby limiting the aggregation and deposition of extracellular Aβ plaques [73]. This dual mechanism, which prevents new Aβ generation while promoting the degradation of toxic intracellular species, highlights the therapeutic potential of PROTACs in modulating amyloid pathology and slowing neurodegenerative progression. While promising, TPD approaches have significant challenges for CNS, especially regarding BBB permeability [74]. Many PROTACs are relatively large polar molecules and will not passively diffuse through the BBB [75]. Researchers are investigating various strategies for CNS delivery, including maximizing the properties of the drug molecules, prodrug-based approaches, and nanoparticle-based drug carriers [76]. Other challenges that may arise are selectivity and off-target degradation of proteins that are not pathogenic, as this could lead to unwanted toxicity [77]. Additionally, developing a clearer understanding of the localized expression of neuronal E3 ligases and the dynamic expression changes over disease states is important for the design of TPD [78]. Finally, pharmacokinetic and metabolic stability concerns may further complicate the development of TPD for the CNS, further complicating dosing regimen and formulation considerations [79]. In conclusion, TPD technologies, specifically PROTACs and molecular glues, demonstrate significant potential for CNS-targeted therapeutics via specific degradation of pathogenic proteins [80]. Nevertheless, effective translatability into treatments for neurodegenerative diseases requires solutions to key obstacles in the following: the efficiency of delivery to the brain, ligase selection, and safety [81]. Thus, combining TPD technologies with complementary strategies, natural product scaffolds, and/or co-therapies using antioxidants provides an enticing direction for further exploration. A summary of targeted protein degradation (TPD) technologies, including PROTACs, molecular glues, and related modalities, is presented in Table 3.

## 5. Rationale for Integrating Antioxidants with PROTACs

### 5.1. Complementary Mechanisms: Oxidative Stress Reduction and Protein Clearance

AD is a multifactorial disorder that exhibits aspects of proteinopathy and oxidative stress. The aggregation of misfolded proteins, including amyloid-β (Aβ) and hyperphosphorylated tau, induces synaptic dysfunction, mitochondrial failure, and chronic neuroinflammation [87]. At the same time, oxidative stress exacerbates protein aggregation, which leads to lipid peroxidation and DNA damage and culminates in neuronal apoptosis. These interrelated pathophysiological phenomena lead to a cycle of self-reinforcement, establishing that any efficacious therapeutic approach must effectively target both mechanisms concurrently [87].

**Figure 6:**
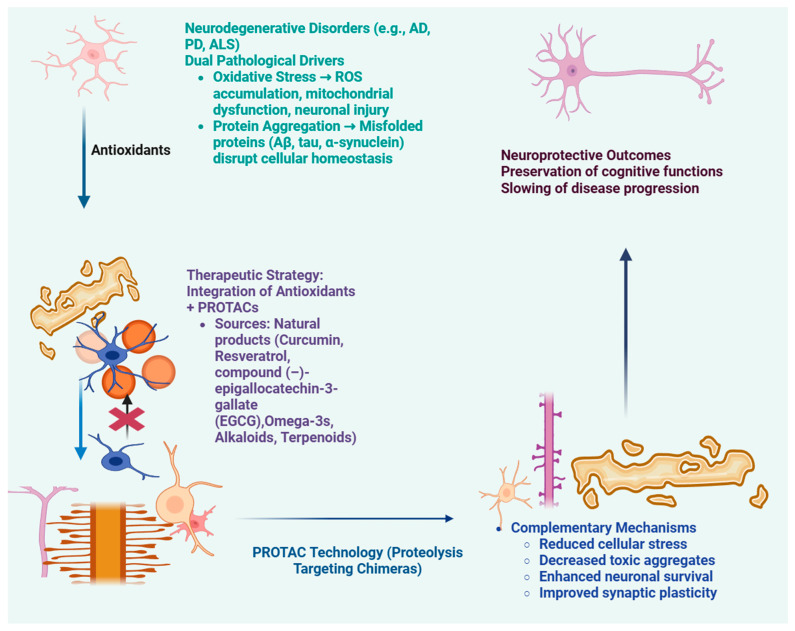
Therapeutic integration of antioxidants and PROTAC technology in neurodegenerative disorders. Neurodegenerative diseases such as Alzheimer’s disease (AD), Parkinson’s disease (PD), and amyotrophic lateral sclerosis (ALS) are driven by dual pathological processes—oxidative stress leading to mitochondrial dysfunction and neuronal injury, and protein aggregation involving misfolded proteins like Aβ, tau, and α-synuclein. Antioxidants derived from natural compounds (e.g., curcumin, resveratrol, EGCG, omega-3s, alkaloids, and terpenoids) help counter oxidative stress, while PROTAC (Proteolysis-Targeting Chimera) technology promotes selective degradation of toxic proteins. The integration of these therapeutic strategies provides complementary mechanisms, including reduced cellular stress, decreased toxic aggregates, enhanced neuronal survival, and improved synaptic plasticity, ultimately leading to neuroprotection, preservation of cognitive function, and slowed disease progression. The rationale for integrating antioxidants with PROTACs, focusing on complementary mechanisms like oxidative stress reduction and protein clearance, is shown in Table 4. (Created in BioRender. Singh, D. (2025) https://BioRender.com/pzlt3uv; accessed on 29 October 2025).

Antioxidant natural compounds and targeted protein degradation (TPD) technologies both represent complementary approaches for this dual intervention. Natural antioxidants scavenge ROS, modulate the Nrf2 pathway, restore mitochondrial function, and reduce neuroinflammation [88]. PROTACs, on the other hand, selectively target the degradation of pathogenic proteins through E3 ligase recruitment and remove toxic protein species responsible for neuronal dysfunction [89]. Each approach can then be combined to provide synergistic actions, with drug action targeting oxidative stress, while simultaneously, the primary protein pathology is directly removed [90]. This dual-action intervention, in theory, will slow or reverse neurodegeneration more reliably than either intervention on their own, targeting both upstream triggers of disease and downstream consequences of AD pathology (Figure 6) [91].

**Table 4:** The rationale for integrating antioxidants with PROTACs focuses on complementary mechanisms like oxidative stress reduction and protein clearance.

S.N.	Integration Aspect	Antioxidant Function	PROTAC Function	Complementary Effects/Rationale	Potential Targets in AD	Notes/References	References
1	Oxidative Stress Mitigation	Scavenge ROS, upregulate Nrf2/ARE pathway, reduce lipid peroxidation, restore mitochondrial function	Facilitates the removal of ROS-generating misfolded proteins indirectly by clearing their source	Reduces neuronal damage caused by oxidative stress while PROTACs remove proteins that exacerbate ROS production	Aβ aggregates, hyperphosphorylated tau, and APP C-terminal fragments	Antioxidants like quercetin, curcumin, and resveratrol can be incorporated into hybrid PROTAC designs	[92]
2	Protein Clearance	Some antioxidants inhibit aggregation (e.g., curcumin, EGCG), but do not remove proteins	Catalytically degrade pathogenic proteins via E3 ligase recruitment and ubiquitin–proteasome pathway	Combination ensures both inhibition of new aggregate formation and removal of existing aggregates	Tau, Aβ, APP C-terminal fragments, α-synuclein	Dual-action approach addresses upstream (oxidative stress) and downstream (proteinopathy) mechanisms	[93]
3	Anti-Inflammatory Effects	Suppresses microglial activation and pro-inflammatory cytokines (TNF-α, IL-1β)	Can target proteins driving inflammatory signaling (e.g., NF-κB, NLRP3 inflammasome components)	Synergistic reduction of chronic neuroinflammation in AD, protecting neuronal networks	NF-κB, NLRP3, pro-inflammatory mediators	Provides multi-level protection against AD progression	[94]
4	Mitochondrial Protection	Maintains membrane potential, ATP production, and biogenesis	Removes proteins that impair mitochondrial function (e.g., tau aggregates)	Preserves energy metabolism and reduces apoptosis; enhances neuronal survival	Tau, misfolded mitochondrial proteins	Integration enhances overall neuronal resilience	[95]
5	Multi-Targeted Neuroprotection	Broad pleiotropic effects: ROS scavenging, anti-inflammatory, mitochondrial support	Selective protein degradation with catalytic efficiency	Combines pleiotropic neuroprotection with targeted clearance, potentially reducing required dosages and off-target effects	Multiple AD-related proteins and pathways	Forms the conceptual basis for “Antiox-PROTACs”	[96]

### 5.2. Putting Potential Targets into the Equation: Tau, Aβ, APP CTFs, and Neuroinflammatory Mediators

The choice of molecular targets is important for the development of an integrated antioxidant-PROTAC strategy [97]. The tau protein, which aggregates into neurofibrillary tangles, is a primary target for therapeutics [97]. PROTACs could be designed to degrade hyperphosphorylated tau, while antioxidants would work to mitigate the oxidative injury that incites the misfolding of tau [98]. Further, Aβ peptides and APP CTFs have been shown to promote plaque formation and toxicity at the synapse. The clearance of these species by PROTACs and the protection of mitochondria by antioxidants may, therefore, additionally mitigate neuronal injury and cognitive decline [99].

Neuroinflammation is also a significant factor in AD progression, as activated microglia and astrocytes release pro-inflammatory cytokines such as TNF-α, IL-1β, and IL-6 [99]. Antioxidants inhibit such inflammatory mediators, and PROTACs can be developed to degrade key signaling proteins that are promoting the chronic inflammatory state, including components of the NF-κB family and NLRP3 inflammasome subunits [100]. This could provide a mechanism for multi-targeted disease modification by addressing both protein aggregation and inflammatory pathways at multi-levels of treatment, addressing cytotoxic triggers and the cellular environment sustaining neurodegeneration [101].

### 5.3. Natural Compounds Like Scaffolds or Warheads for PROTAC Design

Natural antioxidants not only possess therapeutic ability but also serve as molecular scaffolds or warheads for PROTAC design [102]. PROTACs typically contain a ligand for the target protein, a ligand for an E3 ligase, and a chemical linker [103]. Incorporating a natural antioxidant moiety as the target-binding component has several advantages [104]. The first advantage is that these compounds often have intrinsic neuroprotective activity, exhibiting additional clinical benefits apart from protein degradation [105]. The second advantage is that natural compounds have greater structural diversity, creating opportunities to engage previously ‘undruggable’ targets, such as protein aggregates and transcriptional regulators of oxidative stress [106].

For instance, curcumin or resveratrol, which are polyphenols, could act as tau- or Aβ-targeting ligands while simultaneously exerting ROS-scavenging and anti-inflammatory effects [106]. Flavonoids such as quercetin or EGCG could also be used as warheads to recruit E3 ligases to key pathological proteins, enhancing selectivity and limiting degradation of off-target proteins [106]. Because PROTACs employ these natural product scaffolds, PROTACs can incorporate all of the pleiotropic benefits of antioxidants combined with the catalytic efficiency of targeted protein degradation to develop multifunctional therapeutics uniquely designed for complex CNS disorders [107]. These findings suggest that PROTACs, by recruiting E3 ligases to specifically degrade intracellular Aβ oligomers or Aβ-producing enzymes, could not only mitigate intracellular toxicity but also diminish extracellular plaque deposition by limiting aggregation precursors and enhancing proteasomal clearance pathways.

## 6. Combination Delivery and Synergistic Approaches

The successful implementation of antioxidant-based PROTAC approaches necessitates the consideration of delivery, as well as interacting synergistically [108]. Combination delivery systems, such as nanoparticles, liposomes, or polymeric carriers, can help deliver the PROTAC molecule and the antioxidant compounds across the BBB, permitting their action at the neuronal sites of interest at the same time [109]. This type of delivery platform may also enhance pharmacokinetics, CNS penetration, and controlled release for maximum therapeutic benefit and minimal systemic exposure [110]. Synergy can also be realized through time or space coordination. For example, antioxidants may potentiate the neuronal environment by decreasing oxidative stress and inflammation that create a “neuroprotective” environment for protein clearance mediated by PROTAC [111]. In contrast, the PROTAC-mediated degradation of misfolded proteins could decrease ROS generation and mitochondrial dysfunction, providing further neuroprotection from oxidative stress [112]. Computer modeling and high-throughput screening methods can be used to determine the best combinations of compounds, linker length, and E3 ligase specificities for optimal therapeutic synergy [113].

In addition, if a compound integrates both antioxidant and PROTAC properties, it may lower the necessary doses of each ingredient, with a reduction in toxicity that can occur with high doses of either PROTACs or polyphenols [114]. A multi-targeted strategy is especially pertinent for AD, where monotherapy strategies have mostly failed due to the heterogeneity and complexity of the disease [115]. Antioxidant-PROTAC therapeutics would target proteinopathy, oxidative stress, mitochondrial dysfunction, and neuroinflammation simultaneously. This kind of therapy could lead to comprehensive disease modification, leading to an improvement in structural and functional measures in a patient [116].

The combination of natural antioxidant compounds with PROTAC technology presents a new and exciting avenue for the treatment of Alzheimer’s disease [117]. By bringing together various complementary mechanisms, such as the abatement of ROS, anti-inflammatory mechanisms, and targeted clearance of pathological proteins, we are targeting several pathological markers at the same time [118]. Natural compounds not only can act as neuroprotective agents but can also offer a well-designed functional scaffold for PROTAC design that may enhance selectivity, efficacy, and safety [119]. Co-delivery and synergism provide an opportunity to improve therapeutic benefit through these strategies and, as a result, offer a multi-modal therapy to overcome limitations of single-target strategies [120]. Continued advancements in research of both natural product pharmacology and targeted protein degradation will ultimately lead to the emergence of “Antiox-PROTACs,” which may represent a novel next-generation approach for disease-modifying therapy in Alzheimer’s disease, with the potential to significantly improve patient quality of life and outcome [121] (Table 4).

## 7. Discovery and Development Pipeline

### 7.1. High-Throughput Screening of Antioxidant Libraries

The discovery of antioxidant-based therapeutics begins with high-throughput screening (HTS) of natural product libraries, including flavonoids, polyphenols, terpenoids, and alkaloids [121]. Compounds are evaluated for ROS scavenging, protein aggregation inhibition, and neurotoxicity using automated neuronal assays [122,123]. Modern HTS integrates computational tools such as molecular docking and machine learning to prioritize molecules with favorable BBB permeability, physicochemical profiles, and potential for PROTAC scaffold development [124]. This systematic approach enables the identification of candidates with dual antioxidant and protein-targeting properties. Pipeline stages, methodologies, and readouts in preclinical neurotherapeutic studies are shown in Table 5.

### 7.2. In Vitro Assays: ROS Reduction, Aggregation Inhibition, and Neuronal Protection

Following HTS, in vitro assays play a crucial role in validating the mechanistic potential of candidate compounds [131]. ROS reduction can be assessed using probes such as DCFDA or MitoSOX in neuronal cell lines and primary neurons [132]. Protein aggregation inhibition is evaluated through thioflavin T binding, electron microscopy, and filter-trap assays to measure amyloid-β and tau fibrillization [133]. Neuroprotection is further confirmed via assays for cell viability, mitochondrial depolarization, and synaptic integrity markers [134]. These combined outputs help identify compounds with pleiotropic neuroprotective actions, vital for integration into PROTAC-based strategies [134].

### 7.3. Identifying Targets Using Proteomic and Metabolomic Profiling

Proteomic and metabolomic profiling facilitate the selection of compounds for PROTAC design by revealing molecular targets, pathways, and downstream effects of antioxidant candidates [135]. Mass spectrometry-based proteomics identifies proteins that interact with or are modulated by the compound, including key Alzheimer’s disease targets such as tau, APP C-terminal fragments, and oxidative stress-related enzymes [136,137]. Metabolomics complements this by mapping mitochondrial function, redox balance, and neuroinflammatory metabolites, enabling the rational selection of PROTAC targets and the design of multi-modal therapeutic constructs [137].

### 7.4. Designing Antioxidant-FIRST Protacs: Scaffold Design and Linker Chemistry

The final phase focuses on designing antioxidant-based PROTACs, wherein lead natural products possessing inherent protein-binding ligands (“warheads”) are coupled with E3 ligase-recruiting moieties [138]. Scaffold design aims to optimize binding affinity, selectivity, stability, and BBB permeability while retaining the compound’s antioxidant function [139]. Appropriate linker chemistry ensures spatial alignment between the ligand and E3 ligase to facilitate proximal ubiquitination and proteasomal degradation [140]. Iterative cycles of medicinal chemistry, in silico modeling, and cellular assays refine selectivity, potency, and pharmacokinetic properties [141]. Altogether, integrating HTS, mechanistic validation, omics-guided target discovery, and rational PROTAC design forms a cohesive discovery-to-development pipeline for next-generation neurotherapeutics [142].

Multiple natural antioxidants exhibit meaningful neuroprotective and cognitive benefits in preclinical AD models [143]. Curcumin, a polyphenol from Curcuma longa, reduces Aβ accumulation, tau hyperphosphorylation, and oxidative stress, enhancing learning and memory [144]. Resveratrol improves cognition through SIRT1 and Nrf2 activation, boosting mitochondrial function and reducing ROS [145]. Flavonoids such as quercetin and EGCG exert antioxidant and anti-inflammatory effects by limiting microglial activation [146]. Terpenoids including ginsenosides and bilobalide restore mitochondrial membrane potential, promoting neuronal survival and function [147]. Collectively, these findings highlight antioxidant-based PROTACs as promising multi-targeted therapeutics that mitigate oxidative stress and degrade pathogenic proteins, advancing the translational potential for Alzheimer’s disease therapy.

## 8. Preclinical Evidence and Case Studies

### 8.1. Natural Antioxidant Leads with Cognitive and Neuroprotective Effects

Multiple natural antioxidants have demonstrated significant neuroprotective and cognitive benefits in preclinical models of Alzheimer’s disease (AD) [119]. Curcumin, a polyphenol derived from Curcuma longa, reduces amyloid-β (Aβ) accumulation, inhibits tau hyperphosphorylation, and mitigates oxidative stress, thereby improving learning and memory in rodent models [148]. Resveratrol also enhances cognitive performance in APP-transgenic mice through activation of SIRT1 and Nrf2 signaling pathways, which collectively enhance mitochondrial function and reduce ROS levels [148]. Similarly, flavonoids such as quercetin and epigallocatechin gallate (EGCG) exhibit dual antioxidant and anti-inflammatory actions by limiting microglial activation, leading to improved neuronal health and cognition [149]. Furthermore, terpenoids including ginsenosides and bilobalide restore mitochondrial membrane potential, supporting neuronal survival and functional recovery. Collectively, these findings underscore the therapeutic potential of antioxidant leads as multi-targeted neuroprotective agents [150].

### 8.2. Proof-of-Concept PROTACs in Neurodegenerative Models

Recently, targeted protein degradation technologies, especially PROTACs, have emerged in the study of neurodegeneration in preclinical models. Tau-targeted PROTACs were able to selectively clear hyperphosphorylated tau aggregates in both neuronal cultures and transgenic mice, leading to decreased synaptic toxicity and improved behavioral outcomes [151]. Alpha-synuclein-targeted PROTACs also demonstrated benefits in models of Parkinson’s disease, with the latter promoting the degradation of pathogenic forms of alpha-synuclein while sparing the native form [152]. These proof-of-concept studies demonstrate the feasibility of applying TPD approaches in the CNS while also identifying challenges such as BBB penetrance and cellular delivery for translational applications [137,153].

### 8.3. Initial Integrative Efforts: Antioxidant Derivatives in TPD Research

Research is now exploring the potential for natural antioxidant scaffolds to be integrated into TPD modalities [154]. More specifically, polyphenol-derived compounds, with structural alterations, have been studied as potential ligands or warheads for PROTAC constructs, with a view to achieving both antioxidant activity and protein degradation in a targeted manner [155]. Overall, in vitro studies demonstrate that these conjugates can mitigate oxidative stress while promoting ubiquitin–proteasome clearance of aggregation-prone proteins. While full-fledged antioxidant-based PROTACs for AD are still several years from development, early studies validate that natural compounds can serve a dual purpose (i.e., neuroprotection and selective protein clearance) [156].Although the Antiox-PROTAC concept remains largely theoretical, preliminary studies emerging from the field support its feasibility. A few in vitro reports have demonstrated PROTAC-like chimeras that integrate antioxidant scaffolds with E3-ligase ligands to promote degradation of pro-oxide or misfolded proteins. For instance, curcumin-based PROTACs targeting NF-κB and STAT3 have shown effective suppression of inflammatory and oxidative pathways in cancer and neuronal cell models. Resveratrol-derived PROTACs have been designed to modulate the Keap1–Nrf2 signaling pathway, leading to enhanced antioxidant responses and decreased ROS accumulation. Similarly, melatonin-based hybrid degraders have demonstrated both ROS-scavenging potential and proteasome-dependent clearance of aggregation-prone proteins. These pilot findings indicate that antioxidant–PROTAC conjugates could achieve dual functionality, restore redox balance, and degrade pathogenic proteins, thereby supporting further preclinical investigation in neurodegenerative disease models. All the preclinical data support the rationale for combined Antiox-PROTAC approaches, showing that antioxidant agents can have beneficial effects while also informing the development of targeted protein degraders [137]. Future studies will need to focus on optimizing CNS pharmacokinetics, delivery, and selectivity to ultimately translate these approaches to the clinic for treatment of AD and other neurodegenerative disorders. Preclinical evidence of natural antioxidants with cognitive and neuroprotective effects in Alzheimer’s disease models is summarized in Table 6.

## 9. Translational and Clinical Perspectives

### 9.1. BBB Delivery and Brain Bioavailability Challenges

A primary barrier to the clinical translation of antioxidant-based PROTACs is delivering them from systemic circulation across the BBB. Consequently, many PROTACs are polar and/or large molecules that are likely to have very limited passive diffusion [165]. Many natural antioxidants are weakly absorbed after oral dosing or undergo rapid metabolism and undergo chemical modification [166]. Proposed methods for mitigating these obstacles include efforts in prodrug design, lipidation, nanoparticle encapsulation to the desired sites, and receptor-mediated transport systems [167]. Each of these strategies attempts to promote penetration into the CNS at effective concentrations and duration of therapy at the target site of action. Further, to optimize brain delivery, PROTAC molecular size, lipophilicity, and attachment of a linker molecule should be optimized in combinations that yield effects on PROTAC BBB permeability and performance as an antioxidant [168].

### 9.2. Safety, Off-Target Effects, and Metabolic Stability

Safety considerations continue to be an important consideration for any CNS-targeted TPD intervention [169]. Notably, while PROTACS may degrade target proteins, they can also degrade off-target proteins, disrupting critical neuronal or even systemic pathways [170]. Antioxidant-PROTAC conjugates will need to be demonstrated to be non-cytotoxic, not perturb mitochondrial function, and not disrupt normal proteostasis [171]. Furthermore, rapid degradation or biotransformation of the PROTAC or the antioxidant moiety could also impact efficacy and may produce undesirable metabolites [172]. Therefore, preclinical toxicology studies and rational medicinal chemistry modification studies will need to be completed to reduce unintended effects while maintaining the necessary pharmacological activities.

### 9.3. Regulatory and Manufacturing Considerations

Moving antioxidant-PROTAC therapeutics from the bench to the bedside necessitates careful consideration of regulatory and manufacturing considerations [173]. TPD molecules are new chemical entities and thus should be characterized preclinically for pharmacodynamics, pharmacokinetics, and safety considerations. Manufacturing components must also have good reproducibility/purity/stability, especially if natural product scaffolds are sustained, as these products could have variations across different batches [174]. Depending on the CNS delivery, combination treatment strategies, or first-in-class mechanism of action, additional assessments may be warranted by regulatory agencies [175]. This work highlights the importance of chemists, pharmacologists, and clinical team members collaboratively working together to successfully navigate regulatory, scalable, and GMP-compliant manufacturing processes [176]. In conclusion, antioxidant-based PROTACs represent a promising multi-targeted strategy for Alzheimer’s disease, but the success of clinical translation will be determined by several factors, including overcoming challenges associated with the BBB and bioavailability, addressing safety and metabolic stability, determining appropriate biomarker monitoring, and solving regulatory and manufacturing challenges [177]. These factors provide a framework for evaluating next-generation therapeutics from preclinical models to human clinical trials.

## 10. Future Directions and Opportunities

### 10.1. Hybrid Molecules: Antioxidant-PROTAC Chimeras

One area of next-generation AD therapeutics could involve the development of hybrid molecules combining antioxidant scaffolds with PROTAC function [178]. These chimeras can neutralize reactive oxygen species (ROS), while also selectively degrading tau or amyloid-β proteins. A hybrid design combining both mechanisms will maximize therapeutic activity, decrease our need for compounds, and possibly minimize overall toxicity [179]. Natural product-derived model PROTACs retain both antioxidant and protein-targeting activity. Early experimental models suggest that they can be developed for multi-targeted therapeutic interventions in neurodegeneration [180].

### 10.2. Delivery Mechanism: Nanoparticle- and Lipid-Based Systems

Advanced delivery mechanisms will be important for clinical translation. Nanoparticle- and lipid-based delivery strategies can overcome BBB restrictions and optimize bioavailability of a potentially large and/or polar delivery cargo [181]. Encapsulation can help protect antioxidant and PROTAC moieties from metabolic degradation, help to control release, and improve CNS penetration [182]. Targeted delivery through the use of surface ligands or receptor-mediated transport mechanisms can provide increased neuronal uptake, limited off-target effects, and improved pharmacokinetics [183]. This type of delivery platform can also co-deliver more than one therapeutic agent, facilitating the exploration of synergistic and combination approaches to AD therapeutics [184].

### 10.3. Individualized Medicine Approach: Factors in Patient-Specific Pathology

Potentially, patients with AD have high inter-subject heterogeneity with respect to protein pathology, oxidative stress, and neuroinflammatory pathology. Individualized medicine could lead to the tailoring of antioxidant-PROTAC therapies to the individual molecular signatures of patients [185]. Genomic, proteomic, and metabolomic profiling may identify patients most likely to respond to hybrid molecules or combinations of molecular therapeutics [186]. This approach increases the likelihood of success for AD patients based on the compatibility of the therapy with patient-specific pathology and allows precision dosing to avoid complications while achieving favorable cognitive outcomes [187].

### 10.4. Collaboration with Emerging Therapeutics: Gene Therapy and Immunotherapy

Collaboration with emerging therapeutic modalities provides additional opportunities for improved efficacy in AD therapeutics [188]. Antioxidant-PROTAC strategies could be combined with emerging gene therapy strategies such as RNA interference or CRISPR-mediated modulation of tau or APP expression to achieve a synergistic reduction in pathogenic protein load [189]. Similarly, there could be an opportunity for blending with immunotherapy strategies that target concerns around amyloid or tau to enhance clearance, as well as modulate neuroinflammatory responses [190]. Both of these strategies allow multi-faceted approaches and target both the causative proteinopathy as well as subsequent oxidative stress/inflammatory stress [191]. In summary, the future directions in AD therapy are likely to include hybrid-molecule strategies, expanded CNS-targeted delivery, and personalized treatments in KDs, along with collaboration with other therapeutic modalities [192]. All of these strategies describe a framework, shaped by the evolving understanding of AD, that will provide an adaptable intervention model for next-generation disease-modifying strategies as part of a multi-modal intervention that could lead to improved outcomes in AD [193].

## 11. Conclusions

Alzheimer’s disease (AD) is still a significant global health concern with complex pathologies that include protein aggregation, oxidative stress, mitochondrial dysfunction, and chronic neuroinflammation. Conventional therapies have insufficiently targeted disease progression, emphasizing the need for new, multi-level approaches. Combining antioxidant natural compounds with TPD, especially in the form of PROTACs, is a new way to develop therapeutic interventions in the treatment of AD. Antioxidants from natural products, including flavonoids, polyphenols, terpenoids, and alkaloids, can provide neuroprotective characteristics by scavenging for reactive oxygen species, modulating the Nrf2 signaling pathway, restoring mitochondrial function, and suppressing neuroinflammation. PROTACs, in contrast, can target the selective degradation of pathogenic proteins such as tau, amyloid-β, and APP fragments in a catalytic mode, addressing the main proteinopathies involved in AD.

## Figures and Tables

**Table 1:** Summary of major classes of neuroprotective agents, their representative compounds, underlying mechanisms of action, observed neuroprotective effects, and BBB penetration or bioavailability. Additional notes are included for specific pharmacological features, limitations, or therapeutic considerations, along with relevant references.

S.N.	Class	Representative Compounds	Mechanisms of Action	Neuroprotective Effects	BBB Penetration/Bioavailability	Notes	References
1	Flavonoids	Quercetin, Epigallocatechin gallate (EGCG), Luteolin, Kaempferol Apigenin	ROS scavenging, Nrf2 activation, Anti-inflammatory, Mitochondrial protection	Reduce Aβ aggregation, prevent tau hyperphosphorylation, and improve synaptic plasticity	Moderate; often limited by metabolism; enhanced via nano formulations	Widely studied in AD models; multiple in vitro and in vivo studies	[33]
2	Polyphenols	Curcumin, Resveratrol, Ferulic acid, Catechins	ROS scavenging, Nrf2/ARE pathway, Anti-inflammatory, Protein aggregation inhibition	Reduce oxidative stress, inhibit Aβ fibrillization, improve cognition	Poor oral bioavailability; BBB penetration low; improved by liposomes/nanoparticles	Curcumin and resveratrol are extensively studied; clinical translation limited by bioavailability	[34]
3	Terpenoids	Ginsenosides, Bilobalide, Ursolic acid	Mitochondrial membrane stabilization, Anti-inflammatory, ROS reduction	Enhance neuronal survival, restore mitochondrial function, improve memory	Variable; generally moderate BBB permeability	Ginsenosides improves mitochondrial potential; bilobalide supports synaptic function	[35]
4	Alkaloids	Huperzine A, Berberine, Galantamine	ROS scavenging, Anti-inflammatory, Cholinesterase inhibition	Protect against oxidative damage, enhance cholinergic signaling, and improve cognitive function	Huperzine A: good CNS penetration; Berberine: limited	Huperzine A is clinically approved in some regions; dual antioxidant and enzyme inhibition effects	[36]
5	Carotenoids and Vitamins	Lycopene, Lutein, Vitamin C, Vitamin E, Coenzyme Q10	Lipid peroxidation prevention, ROS scavenging, and Mitochondrial support	Stabilize membranes, reduce oxidative damage, support energy metabolism	Moderate to low BBB permeability; vitamin C and E cross with variable efficiency	Often used as dietary supplements, neuroprotective efficacy has been shown in animal models	[37]
6	Other Natural Products	Sulforaphane, and Curcuminoids	Nrf2 activation, ROS scavenging, Anti-inflammatory	Reduce oxidative stress, improve mitochondrial function, and modulate microglial activation	Variable; sulforaphane shows good CNS availability	Emerging compounds; require further preclinical validation	[38]

**Table 2:** Integration of Antioxidants and PROTAC Pathways in Alzheimer’s Disease Therapy.

Category	Mechanism/Target	Molecular Effect	Therapeutic Outcome	Integration Potential (Antiox–PROTAC Model)
Antioxidant Mechanisms	ROS scavenging (e.g., SOD, catalase, glutathione)	Reduces oxidative stress and lipid peroxidation	Protects neuronal integrity	Antioxidant PROTACs could stabilize redox balance and prevent protein misfolding
Mitochondrial Protection	Activation of Nrf2/ARE pathway	Enhances antioxidant enzyme transcription	Improves mitochondrial function	Nrf2-activating PROTACs may promote clearance of damaged mitochondria
PROTAC Pathways	E3 ligase–mediated ubiquitination	Induces targeted protein degradation	Removes toxic proteins (Aβ, tau)	Enables selective degradation of pathogenic aggregates
Integration Models	Dual-acting Antiox–PROTAC conjugates	Combines antioxidant and degradation functions	Reduces Aβ load and oxidative damage	Provides synergistic neuroprotection and disease modification
Translational Outlook	In vitro to in vivo validation	Assesses efficacy, PK/PD, BBB permeability	Supports therapeutic feasibility	Paves way for hybrid Antiox–PROTAC therapeutics in AD

**Table 3:** Summarizing targeted protein degradation (TPD) technologies, including PROTACs, molecular glues, and related modalities.

S.N.	TPD Modality	Mechanism	Key Features	Representative Targets/Examples	Advantages	Limitations/Challenges	References/Notes	References
1	PROTACs (Proteolysis-Targeting Chimeras)	Bifunctional molecules recruit a target protein to an E3 ubiquitin ligase, leading to ubiquitination and proteasomal degradation	Consist of a target-binding ligand, E3 ligase ligand, and a linker; catalytic mechanism; can degrade “undruggable” proteins	Tau, Amyloid-β, α-synuclein, BRD4, BCL-2	Sub-stoichiometric dosing, high selectivity, target protein clearance rather than inhibition	Large molecular weight; poor BBB permeability; linker optimization critical; potential off-target degradation	Widely used in oncology; CNS applications emerging; hybrid designs with natural products are under exploration	[82]
2	Molecular Glues	Small molecules stabilize the interaction between an E3 ligase and target protein, leading to degradation	Single, small-molecule drug; does not require linker; often discovered serendipitously	IKZF1/3 (thalidomide, lenalidomide), CDK12, GSPT1	Simplified chemistry compared to PROTACs; can engage proteins lacking high-affinity ligands	Target discovery challenging; requires compatible E3 ligase interface; off-target effects possible	FDA-approved examples exist (thalidomide derivatives); CNS applications limited	[83]
3	LYTACs (Lysosome-Targeting Chimeras)	Direct extracellular or membrane proteins to lysosomes via receptor-mediated endocytosis	Uses glycan-based ligands to engage lysosomal trafficking receptors	EGFR, PD-L1	Enables degradation of extracellular or membrane proteins not accessible to proteasome	Limited CNS penetration; relatively large molecules; receptor expression dependency	Emerging modality; potential for targeting AD-related extracellular aggregates	[84]
4	AUTACs (Autophagy-Targeting Chimeras)	Tags target proteins for autophagic degradation via K63-linked ubiquitination	Can degrade cytosolic aggregates and damaged organelles	Misfolded tau, damaged mitochondria	Access to larger or aggregated proteins; organelle-targeted degradation	Mechanism still under investigation; slower degradation kinetics than PROTACs	Preclinical stage; potential synergy with antioxidant pathways	[85]
5	ATTECs (Autophagosome-Tethering Compounds)	Tethers target proteins directly to autophagosomes for selective autophagy	Does not rely on ubiquitination; small molecule tether	mHTT (Huntingtin), aggregated tau	Can target aggregated or insoluble proteins	Early development: CNS delivery remains a challenge	Demonstrated in Huntington’s and AD models; proof-of-concept studies	[86]

**Table 5:** Pipeline Stages, Methodologies, and Readouts in Preclinical Neurotherapeutic Studies.

S.N.	Pipeline Stage	Description/Methodology	Readouts/Assays	Purpose/Outcome	Examples/Notes	References
1	Library Selection	Compilation of natural product libraries rich in antioxidants, including flavonoids, polyphenols, terpenoids, alkaloids, and carotenoids	Chemical diversity assessment, structural classification	Identify a broad pool of candidate compounds with potential neuroprotective activity	Commercial natural product libraries; curated botanical extracts; in-house isolated compounds	[125]
2	High-Throughput Screening (HTS)	Automated screening platforms using multi-well plates, robotics, and fluorescence/luminescence readouts	ROS scavenging (DCFDA, MitoSOX), Aβ/tau aggregation inhibition (ThT binding), cytotoxicity (MTT, LDH release)	Rapid identification of compounds with antioxidant and anti-aggregation potential	384- or 1536-well plate formats; fluorescence-based kinetic assays	[126]
3	Computational Pre-Screening	In silico docking and predictive modeling to assess target engagement, BBB permeability, and ADMET properties	Molecular docking scores, predictive BBB permeability, Lipinski’s rule-of-five, toxicity predictions	Prioritize compounds with favorable pharmacokinetics and CNS bioavailability before experimental screening	Software: AutoDock (4.2.6), Schrödinger (2025-4), ADMET predictor 11.0; reduces experimental load	[127]
4	Hit Identification	Selection of compounds meeting threshold activity in primary HTS	ROS inhibition > 50%, aggregation inhibition > 50%, low cytotoxicity	Leads are advanced for mechanistic validation	Hits often include curcumin, EGCG, quercetin, resveratrol, ginsenosides	[128]
5	Secondary Validation	Dose–response studies, orthogonal assays, and target specificity confirmation	Concentration-dependent ROS reduction, aggregation kinetics, neuronal viability, mitochondrial assays	Confirm reproducibility and specificity of hits; eliminate false positives	Multiple cell lines, including primary neurons, SH-SY5Y cells, and iPSC-derived neurons	[129]
5	Mechanistic Profiling	Investigation of pathways engaged by lead compounds	Nrf2/ARE activation, anti-inflammatory signaling, mitochondrial function assays	Determine pleiotropic neuroprotective mechanisms to guide further development	Western blot, qPCR, ROS imaging, Seahorse mitochondrial analysis	[130]
6	Lead Selection for PROTAC Development	Integration of chemical and biological data to select compounds suitable as PROTAC warheads or scaffolds	Consider BBB penetration, potency, target engagement, and antioxidant activity	Identify candidates for hybrid antioxidant-PROTAC design	Structural features (hydroxyls, phenolic groups) may facilitate linker attachment	[130]

**Table 6:** Summary of preclinical evidence of natural antioxidant leads with cognitive and neuroprotective effects in Alzheimer’s disease models.

SN.	Natural Compound	Class	Model Systems	Mechanisms of Action	Neuroprotective/Cognitive Effects	BBB Penetration/Bioavailability	Notes	References
1	Curcumin	Polyphenol	Transgenic AD mice (APP/PS1), primary neurons	ROS scavenging, Nrf2 activation, inhibition of Aβ aggregation, tau hyperphosphorylation suppression	Reduces amyloid plaques, decreases tau phosphorylation, and improves spatial learning and memory	Poor oral bioavailability; enhanced by nano formulations or liposomes	Widely studied; dual antioxidant and anti-aggregation activity	[157]
2	Resveratrol	Polyphenol	Tg2576 mice, SH-SY5Y cells	Activates SIRT1, Nrf2 pathway, mitochondrial biogenesis, and anti-inflammatory	Reduces oxidative stress, improves synaptic plasticity, and enhances learning and memory	Low bioavailability; CNS penetration moderate; nanoencapsulation improves delivery	Neuroprotective and anti-inflammatory; synergistic with other polyphenols	[158]
3	Quercetin	Flavonoid	3xTg-AD mice, primary cortical neurons	ROS scavenging, Nrf2 activation, anti-inflammatory, inhibition of Aβ aggregation	Improves memory performance, reduces neuroinflammation, and prevents neuronal apoptosis	Moderate BBB permeability; metabolized rapidly	Used in combination with other flavonoids for enhanced efficacy	[159]
4	Epigallocatechin Gallate (EGCG)	Flavonoid	APP/PS1 mice, SH-SY5Y cells	Antioxidative, inhibits Aβ fibrillization, anti-inflammatory, and mitochondrial protection	Reduces amyloid plaques, preserves synaptic function, enhances cognition	Moderate; low oral bioavailability; improved via nanoparticles	Green tea polyphenol: well-documented neuroprotective effects	[160]
5	Ginsenosides (Rb1, Rg1)	Terpenoid	AD rat and mouse models	Mitochondrial membrane stabilization, ROS reduction, neurotrophic signaling	Enhances neuronal survival, improves learning/memory, restores mitochondrial function	Moderate; variable depending on compound; can cross BBB	Show multi-targeted neuroprotection; often used in combination with other compounds	[161]
6	Huperzine A	Alkaloid	APP/PS1 mice, scopolamine-induced memory impairment	ROS scavenging, acetylcholinesterase inhibition, and anti-inflammatory	Enhances cholinergic signaling, protects neurons, and improves spatial memory	Good CNS penetration; clinically approved in some regions	Dual action: antioxidant and enzyme inhibition	[162]
7	Vitamin E (α-tocopherol)	Vitamin/Carotenoid	APP/PS1 mice, aged rats	Lipid peroxidation prevention, ROS scavenging	Reduces oxidative stress, slows cognitive decline	Moderate; crosses BBB	Widely used in supplementation studies; often combined with other antioxidants	[163]
8	Coenzyme Q10	Quinone	Transgenic AD mice, primary neurons	Mitochondrial support, ROS scavenging	Preserves mitochondrial function, reduces neuronal apoptosis, and improves cognition	Moderate; CNS penetration limited; improved by formulations	Supports energy metabolism; synergistic with other antioxidants	[164]

## Data Availability

No new data were created or analyzed in this study. Data sharing is not applicable to this article.
